# Talking about premature ejaculation in primary care: the GET UP cluster randomised controlled trial

**DOI:** 10.3399/BJGPO.2021.0168

**Published:** 2022-03-09

**Authors:** Marie Barais, Marine Costa, Camille Montalvo, Vincent Rannou, Hélène Vaillant-Roussel, David Costa, Sébastien Cadier, Bruno Pereira

**Affiliations:** 1 Department of General Practice, Université de Bretagne Occidentale, Brest, France; 2 Department of General Practice, Faculty of Medicine, Clermont Auvergne University, Clermont-Ferrand, France; 3 Department of Clinical Research and Innovation, Clermont-Ferrand University Hospital, Clermont-Ferrand, France; 4 Département Universitaire de Médecine Générale, UFR Médecine Université de Montpellier-Nimes, Montpellier, France; 5 Département de Médecine Générale, UFR Sciences Médicales, Université de Bordeaux, Bordeaux, France

**Keywords:** communication, male, premature ejaculation, referral and consultation, sexual dysfunction, general practice, primary healthcare

## Abstract

**Background:**

Premature ejaculation (PE) is the most common sexual dysfunction in males. A previous qualitative study identified six communication strategies described by GPs to tackle this topic during consultations.

**Aim:**

To determine whether these six strategies are more effective than usual care for promoting discussion about PE between patients and their GPs.

**Design and setting:**

Cluster randomised controlled trial, stratified in four French regions, with an intervention group (GPs who received a training session on the six communication strategies) and a control group (routine medical care). Participants were males aged 18–80 years consulting for a sexual, urogenital, or psychological reason.

**Method:**

The efficacy of the training session in communication skills, compared with usual care, was evaluated by determining the percentage of patients who discussed PE with their GP (primary outcome). The percentage of enrolled patients with PE was calculated using a cut-off score >9 of the premature ejaculation diagnostic tool (PEDT) completed by the enrolled patients at Week 4 after the consultation. The quality-of-life changes were evaluated as the SF-12 scale score difference between baseline and Week 4 post-consultation.

**Results:**

In total, 130 patients were included by 32 GPs (*n* = 16 in the intervention and *n* = 16 in the control group). The percentage of patients who discussed PE was higher in the intervention group than in the control group (42.0% versus 4.9%, absolute difference = 37.1%; 95% confidence intervals [CI] = 24% to 50%, *P*<0.001).

**Conclusion:**

Training GPs in communication strategies about PE improves its detection.

## How this fits in

PE is the most common sexual complaint in males (21–30% of males aged 18–80 years), and GPs are the first health professionals with whom patients discuss PE. PE was discussed more often by patients who consulted GPs who had undergone training on communication strategies on PE (intervention group) than by patients whose GPs did not (control group): 42% versus 4.9% of patients with PE*, P*<0.001. These results indicate that communication strategies on PE are useful in the naturalistic settings of primary care. The quality-of-life scores at baseline and after 1 month were not significantly different in patients in the intervention and control groups.

## Introduction

PE is the most common sexual complaint in men: 21–30% of males aged 18–80 years report low or absent control over ejaculation and/or too early ejaculation.^1–3^ This is often associated with anorgasmia, low libido, depression, and anxiety that affect the patients’ and their partners’ quality of life.^4,5^ The definition of PE by the International Society for Sexual Medicine (ISSM)^6,7^ is an '*ejaculation that always, or nearly always, occurs prior to, or within about one minute of vaginal penetration; the inability to delay ejaculation during all or most vaginal penetrations, with negative personal consequences, such as distress, anxiety, frustration and/or avoidance of sexual intimacy*'. Besides the distinction between acquired and lifelong PE, two other categories have been described: '*variable PE*' and '*subjective PE*'.^8^ Variable PE corresponds to the normal variation in sexual performance. Subjective PE corresponds to a distorted perception of the time before ejaculation: the patient thinks he has PE, although his intravaginal ejaculation latency time (IELT) is longer than 1 minute.

GPs are the first health professionals with whom patients discuss PE.^9^ A cross-sectional survey based on structured questionnaires completed by >300 males in German family practices’ waiting rooms found that most males considered it important to talk with their GP about their sexual concerns.^10^ However, almost half of them wished that their GP would initiate the discussions about sexuality. More than two-thirds of responders would have liked their GP to signal his/her open-mindedness by directly addressing sexual topics during the consultation.^10^ Moreover, 80% of males who participated in the aforementioned study said that they had already experienced, at least occasionally, a sexual problem. However, only 12% of these males consulted their GP about it.^10^ The ISSM guidelines emphasise the GP’s role in PE management:^4^ GPs should '*recognise PE and make patients feel comfortable about getting hel* *p*'.^7^ However, GPs often find it difficult to talk about their patients’ sexual problems, and do not include the taking of sexual history in their routine practice.^11^ The rate of sexual history-taking in primary care is low and depends on the patient’s age and GP’s training level and sex.^12^ Lack of time is the most significant factor.^13–17^ Moreover, GPs often consider sexual dysfunction less important compared with delivering information on/detecting sexually transmitted infections and contraceptive counselling.^13–17^ They also believe that they have insufficient training,^10^ or are ill-qualified to deal with sexual problems.^13,17–19^ GPs often talk about '*opening a can of worms*' when describing their difficulties in addressing sexual dysfunction during consultations.^13,17^

Training in communication skills has been identified as the most important predictor of GPs’ willingness to ask patients about their sexual history.^20^ In 2009, the authors of the present work carried out a qualitative study to identify strategies used by GPs to initiate discussion on PE.^21^ The content analysis of the semi-structured interviews carried out with 11 GPs identified six communication strategies to tackle the subject (described in the Method section). The present study investigated whether training in communication skills based on these six strategies facilitated discussion of PE between GPs and patients compared with usual care (evaluated as the percentage of patients who mentioned PE with their GP).

## Method

### Study design and participants

The GET UP trial was a randomised controlled trial with two parallel clusters (intervention group and control group). The study protocol has already been described and is available at the following link.^22^ GPs and their patients were from four French regions (Brittany, Aquitaine, Massif Central, and Languedoc-Roussillon-Midi-Pyrénées). They did not receive any payment for their participation. GPs and patients were representative (age, sex, and location distribution) of the French population consulting in primary care.^23^ GPs with an exclusive specialty (for example, acupuncture or homeopathy) or with specialised training in sexology and communication skills were not included in the study.

The selected GPs consecutively enrolled males aged 18–80 years who presented a urogenital, sexual, or psychological complaint listed in the International Classification of Primary Care, second edition (ICPC-2). This list was used to guide patient selection. GPs informed the patients about the study after discovering the reason for the visit, at the beginning of the consultation.

Exclusion criteria were patients consulting for other reasons, patients not speaking French, patients with low decision-making capacity (such as cognitive impairment, or severe depression), and patients unable to sign the written informed consent.

GPs’ randomisation was stratified by region and by sex, using block randomisation sequences generated by Stata software (version 13). The GP’s practice was the unit of randomisation. Therefore, all GPs working in the same practice (and their patients) were randomised in the same group (intervention or control) to avoid contamination bias.

GPs in the intervention group and the researchers recruiting the GPs were not blinded to the allocation. However, GPs in the intervention group were asked not to disclose their allocation status to avoid GPs in the control group becoming aware of their control status. Patients were blinded to their GP’s allocation and the trial primary outcome.

### Intervention and control groups

The intervention consisted of an interactive 4-hour workshop to train GPs on the use of the six strategies identified in the qualitative study (intervention group):^22^

- Being receptive during and particularly at the end of the consultation, just before opening the door, to create a pause that the patients could use to voice their problem.
- Using gentle humour to lighten the atmosphere.
- Matter-of-fact approach (natural and mechanical function of sexuality) to reduce the patient’s embarrassment.
- Question on sexual dysfunction during consultations dedicated to sexual health prevention.
- Suggesting some signs and symptoms associated with the current clinical situation (for example, '*you’re showing symptoms of depression, and depression can bring physical, psychological, and even sexual fatigue*') to help the patient to start talking about PE.
- Facilitating the patients’ verbal expression; enquiring about their psychosocial and medical history and daily environment enables them to talk about PE.

GPs assigned to the control group provided care according to their usual practice: '*clinical care without any value* *judgment*' and centred on the patient.^21^ The GPs in the control group attended a 45-minute information session that included a slide presentation to describe the patient inclusion/non-inclusion criteria and the outcome questionnaire. This information session was organised without giving any information on the communication strategies or sexual issues to limit any influence on their usual behaviour.^24^

### Outcomes

The primary outcome was the percentage of patients who talked about PE with their GP during the inclusion consultation in the intervention and control groups. To determine this percentage, GPs in the two groups were asked to fill in the outcome questionnaire after the consultation. In this questionnaire, they specified whether the patient and GP discussed sexual (PE, erectile dysfunction), urinary (dysuria, urinary incontinence), or psychological (anxiety, depression) problems. These different topics were included to avoid contamination bias in the control group. An English version of this questionnaire is available as Supplementary Material.

Secondary outcomes were 1) the percentage of patients with PE, and 2) the patients’ quality-of-life changes between baseline and Week 4 post-consultation. PE was evaluated using the validated French version of the PEDT,^25,26^ completed by the patients at Week 4 post-consultation. This 4-week interval between visit and PEDT completion was chosen to dissociate the diagnosis of PE from the consultation and reduce any hypothetical effect of the consultation on the patient’s perception of his ejaculation. The PEDT is an extensively validated, self-reported measure that uses the Diagnostic and Statistical Manual of Mental Disorders, revised version 4 (DSM-IV-TR) criteria to detect PE.^27^ This brief and simple screening tool is recommended by ISSM.^8–28^ A score ≥9 suggests possible PE, and a score ≥11 indicates the presence of PE.

Quality of life was assessed with the validated French version of the self-reported SF-12 questionnaire,^29^ completed by patients immediately after the consultation (available in the waiting room) and at Week 4 post-consultation (sent back by post). The 4-week interval was chosen to focus mainly on the PE effects on quality of life, anticipating that after a longer interval, other problems could also affect the scores.

### Statistical methods

Hierarchical regression models (that is, generalised linear mixed model for the binary endpoint) were used to estimate the intervention effect on the percentage of patients who discussed PE by taking the inter- and intra-GP variability into account. Intraclass correlation coefficients (ICC) were estimated for each group and the results were expressed as absolute differences and 95% CIs. Multivariate analyses were performed to take into account possible confounding factors: sex, age, and geographical area for GPs, and age and socioeconomic status for patients. The secondary outcomes were analysed according to the protocol.^22^ The SF-12 scores were compared between groups by covariance analysis, with the baseline score as covariate, as suggested by Klar and Darlington.^30^ When the SF-12 score at Week 4 was missing, it was replaced by the baseline score. Patients with complete data were compared with patients lost to follow-up to validate the representativeness of the sample.

The PEDT score was analysed as a quantitative variable with the statistical methods used for the SF-12 score, and as a binary outcome (PE/no PE using the scores ≥11 and ≥9 as cut-offs) with a generalised linear mixed model.

A sensitivity analysis was carried out to analyse the attrition bias and to characterise the statistical nature of missing data. There was no significant difference between the profiles of the missing data and analysed data.

## Results

### GP and patient recruitment

**Figure 1.**
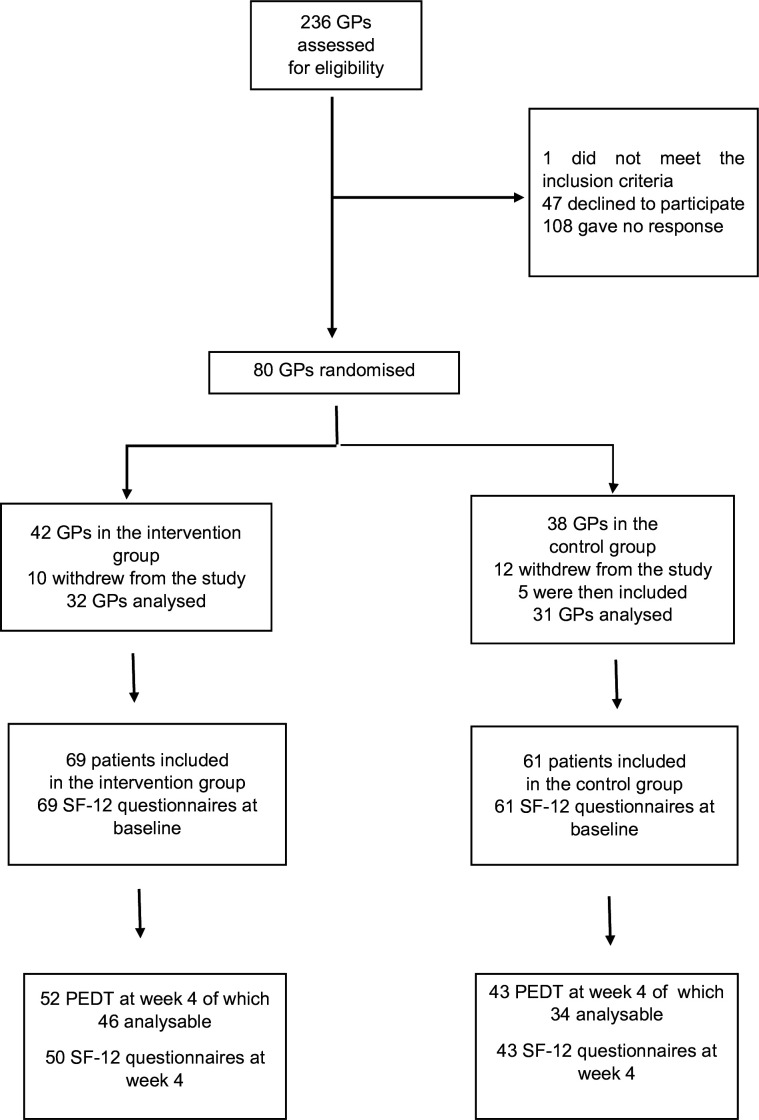
Study flowchart

Among the 236 GPs assessed for eligibility in April 2016 and May 2017, 80 were randomised in the intervention (*n* = 42) and control group (*n* = 38) (Figure 1). Twenty-two GPs withdrew during the study period (*n* = 10 in the intervention group, *n* = 12 in the control group) because they realised that they did not have time for the study. Five additional GPs were recruited and assigned to the control group to have 32 GPs in the intervention group and 31 in the control group. Thirty-two GPs (*n* = 16 in the intervention group, *n* = 16 in the control group) included 130 patients (*n* = 69 in the intervention group and *n* = 61 in the control group).

### Baseline characteristics

The baseline characteristics of the GPs in the intervention (*n* = 32) and control groups (*n* = 31) were comparable (Table 1). They were mainly males and working in urban areas. The patients’ characteristics (age, living with a partner, social characteristics) in the two groups also were not significantly different (Table 1). Their mean age was 58.5 years (standard deviation [SD] = 13.7), approximately 75% of them lived with a partner, and 26% were retired.

**Table 1.** Characteristics of the GPs and patients involved in the study

Characteristics	Total	Intervention	Control	*P* value
**GP** **s** **,** ***n*** ^**a**^	63	32	31	
Age in years, mean (SD)	50.4 (10.1)	50.4 (10.2)	50.4 (10.0)	0.99
Sex male, *n* (%)	37 (58.7)	19 (59.4)	18 (58.1)	0.92
Work settings				
Urban, *n* (%)	38 (60.3)	17 (53.1)	21 (67.7)	0.24
Group practice, *n* (%)	58 (92)	29 (94)	29 (94)	0.99
**GP** **s** **,** ***n*** ^**b**^	32	16	16	
Age in years, mean (SD)	49.1 (10.3)	46.8 (10.4)	51.4 (10.1)	0.22
Sex male, *n* (%)	16 (50.0)	7 (43.8)	9 (56.3)	0.48
Work settings				
Urban, *n* (%)	18 (56.3)	9 (56.3)	9 (56.3)	1.00
Group practice, *n* (%)	31 (96.9)	15 (93.8)	16 (100.0)	1.00
**Patients,** ***n***	130	69	61	
Age in years, mean (SD)	58.5 (13.7)	56.8 (13.7)	60.4 (13.6)	0.13
Living with a partner, *n* (%)	97/128 (75.8)	53/69 (76.8)	44/59 (74.6)	0.77
Employment status, *n* (%)				0.75
Working	79 (60.8)	44 (63.7)	35 (57.4)	
No occupation	8 (6.2)	5 (7.3)	3 (4.9)	
Retired	34 (26.1)	16 (23.2)	19 (29.5)	
No information	9 (6.9)	4 (5.8)	5 (8.2)	

^a^Total number of recruited GPs. ^b^GPs who enrolled patients. SD = standard deviation.

### Primary outcome

The number of patients who talked about PE with their GP during the consultation was significantly higher in the intervention group than in the control group: 29 versus 3 (42% versus 4.9%, absolute difference = 37%, 95% CI = 24% to 50%, *P*<0.001; ICC = 0.25, 0.41, and 0.24 for the whole sample, the control, and the intervention group respectively), as shown in Table 2. Similarly, more patients talked about erectile dysfunction in the intervention than in the control group: 81.2% versus 39.3%, absolute difference = 42%, *P*<0.001 (Table 2).

**Table 2.** Topics discussed during the consultation (outcome questionnaire completed by GPs)

	Total (*n* = 130)	Intervention (*n* = 69)	Control (*n* = 61)	Absolute difference (95% CI)	*P* value(univariate)	*P* value (multivariate)
**S** **exual**						
PE, *n* (%)	32 (24.6)	29 (42.0)	3 (4.9)	37%(24% to 50%)	0.001^a^	<0.001^a^
Erectile dysfunction, *n* (%)	80 (61.5)	56 (81.2)	24 (39.3)	42%(26% to 57%)	0.001^a^	0.003^a^
Urinary						
Dysuria, *n*(%)	87 (66.9)	41 (59.4)	46 (75.4)	–16%(−32% to 0%)	0.055	0.265
Urinary incontinence, *n* (%)	34 (26.2)	15 (21.7)	19 (31.2)	–9%(–25% to 6%)	0.235	0.686
**P** **sychological**						
Anxiety, *n*(%)	76 (58.5)	44 (63.8)	32 (52.5)	11%(–6% to 28%)	0.400	0.042^a^
Depression, *n*(%)	51 (39.2)	29 (42.0)	22 (36.1)	6%(–11% to 23%)	0.518	0.330

^a^Statistically significant. PE = premature ejaculation.

### Secondary outcomes

The SF-12 scores (quality of life) did not significantly change between baseline and Week 4 after the consultation in the intervention group: mean difference = -2.9 (95% CI = -6.1 to 0.4; *P* = 0.08) for physical quality of life; and mean difference = 1.9 (95% CI = -2.1 to 5.5; *P* = 0.35) for mental quality of life. It also did not significantly change between groups (Table 3).

**Table 3.** SF-12 scores at baseline and at month one after the consultation in the intervention and control groups

		BaselineITT/PP	1 monthPP	Mean difference(95% CI)	*P* value
Physical dimension, mean (SD)	Intervention group	47.2 (8.7)/46.4 (9.0)	46.0 (9.3)	–0.4 (–2.5 to 1.6)	0.69
	Control group	44.3 (8.1)/44.6 (8.3)	47.2 (9.6)	2.6 (0.6 to 4.6)	0.01
	Intervention group versus control group			**–2.9 (–6.1** **to 0.4**)	**0.08** ^a^ **0.13** ^b^
Mental dimension, mean (SD)	Intervention group	44.7 (8.2)/45.1 (7.6)	45.1 (9.0)	0.0 (–2.5 to 2.5)	0.98
	Control group	46.8 (10.5)/47.6 (9.3)	44.8 (9.4)	–2.7 (–5.6 to 13.0)	0.06
	Intervention group versus control group			**1.9 (–2.1** **to 5.5**)	**0.35** ^a^ **0.29** ^b^

^a^PP using the imputation method; that is, the baseline score was used to replace the score at Week 4 when missing. ^b^ITT using the imputation method.

ITT = intention-to-treat analysis. PP = per-protocol analysis. SD = standard deviation.

**Figure 2.**
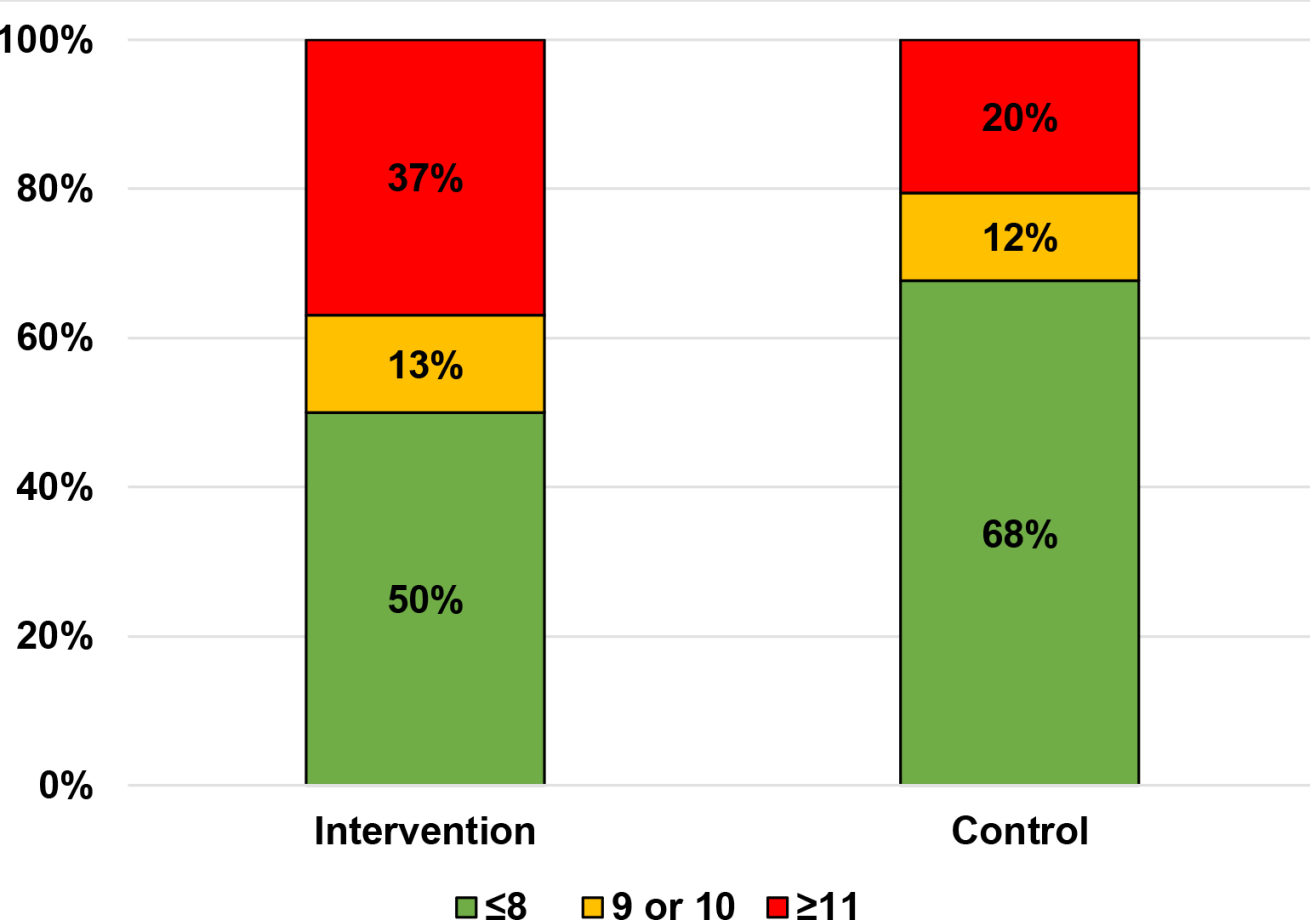
PEDT scores at Week 4 after the consultation, where a score of ≤8 signifies no PE; 9–10 signifies probable PE; and ≥11 signifies PE (*P* = 0.24 between groups)

Of the PEDTs completed at Week 4, 46 could be analysed in the intervention group and 34 could be analysed in the control group (Figure 2). Using the PEDT cut-off of 11, 37.0% (*n* = 17) of patients in the intervention group and 20.6% (*n* = 7) of patients in the control group had PE. Using the cut-off of 9, 42.5% of patients had PE (intervention: *n* = 23, control: *n* = 11). In total, 47.8% (*n* = 11/23) of patients with a score ≥9 in the intervention group talked about PE with their GPs, but none did in the control group. In total, 66.7% of patients with PEDT score ≥11 (*n* = 16/24; intervention: *n* = 9, control: *n* = 7) did not talk about PE with their GP.

## Discussion

### Summary

PE was discussed more often by patients consulting GPs in the intervention than consulting GPs in the control group (42% versus 5% of patients with PE, *P*<0.001). This indicates that communication strategies on PE are useful in the naturalistic settings of primary care. GPs can use them to more readily identify patients with PE and offer adapted care.

Concerning the secondary outcomes, the quality-of-life scores at baseline and after 1 month were not significantly different.

### Strengths and limitations

One of the strengths of this study is that the cluster design was well suited to the organisation of primary care in France. Moreover, contamination bias was avoided by allocating all GPs from the same practice in the same group. GPs in the control group did not know they were in this group, so that it would not affect their behaviour.^31^ The ICC was 0.25, meaning that the human factor role was higher than 20%. The complexity of the GP–patient relationship in primary care consultations, which is the basis of patient-centred care, was respected by the naturalistic context of the study. The six communication strategies were integrated in the French GPs’ consultation organisation. The mean length of a GP’s consultation is 16.4 minutes in France,^32^ compared with 11.7 minutes in the UK.^33^ French patients pay directly at the end of the consultation and are then reimbursed by social security, and they also choose their practitioner. French GPs generally do the administrative work (for example, signing prescriptions, sending the information to social security for reimbursement) during the consultation. Although the French and English healthcare systems are different, the six communicative strategies concern the core of the consultation and could, therefore, be easily integrated in any primary care system.

The major limitation was the small number of participating GPs, owing to the withdrawal of 22 GPs during the study, mainly because of lack of time to devote to the study. Moreover, the 63 GPs participating in the study included only 130 patients — a much lower number than the 600 initially estimated in the sample size calculation. GP recruitment was performed through personal contacts, by targeting friendly networks,^34^ and by informing and presenting the study using different media. Moreover, several personal emails with positive messages were sent to encourage GPs who had not included any patients yet. Participation in research projects is very uncommon among French GPs. A 2019 study of French GPs found that only half of those surveyed were interested in participating in research as primary investigators, with lack of time (79.4%) and administrative burden (43.6%) being the main reasons for non-participation.^35^ Including patients and filling in questionnaires represented additional and non-routine work. These reasons motivated a recruitment through personal contacts to include GPs personally engaged in research. This might be a limitation, but without this strategy a lower rate of participation was anticipated, and thus insufficient statistical power to detect any effect.

The results are also limited by the small number of patients recruited during the study, among whom only 34 patients had a PEDT score ≥9. This small number could also explain the absence of quality-of-life score changes between baseline and Week 4. For this study, patients were not asked to specify their sexual orientation, and this might limit the generalisability of the results. It should be noted that the ISSM definition of PE takes into account only heterosexual and bisexual cis males because it refers only to vaginal penetration.^8^ Similarly, no information was collected on patients' number of partners, sexual intercourse frequency, or history of sexually transmitted infections. Moreover, the GPs did not enquire whether patients talked about these issues with them at their partner’s or partners’ suggestion. This lack of data concerning the patients’ sexual history is another study limitation.

### Comparison with existing literature

Many drugs have been studied for PE management.^36^ The IELT is the reference to compare the different treatments,^37^ but it is difficult to use in real practice. Repeated IELT measurements may deter many males from participating in studies, and self-reported measure of ejaculatory latency has greater ecological validity.^38^ In naturalistic conditions and in primary care, discussing the feelings and consequences of PE on the life of the patients and their partners takes precedence over discussion on the IELT. GPs are essential in PE care, but no recommendation is available on how they should proceed to introduce the topic.^8,39^ Focusing on IELT and putting the emphasis on this information could reinforce the burden of needing to 'last' during sexual intercourse.^40,41^ The focus on the patients and not on their pathology is one of the GPs’ core competencies. Therefore, in this study the 1-minute criterion or any other timeframe was not used to define PE. The patients’ distress caused by PE (or by what they perceived to be PE) was more important than the theoretical compliance with the PE definition. For the same reason, the self-report PEDT was chosen because it is brief and easy, without a set timeframe.

Finally, these communication strategies are not specific to PE and could be useful for initiating discussion on other sensitive topics. This concrete step of 'how to do something in practice and how to be sure that it is efficient' is often lacking in primary care. This study provides concrete communication strategies to initiate discussion in this area.

### Implications for practice

The investigation of an intervention involving six communication strategies and the development of a sexual health communication tool for use in primary care could meet the needs of both practitioners and patients. Implementing these strategies in real practice provides the main added value of this study. The ISSM guidelines state that GPs have an important role to play in PE diagnosis and treatment, and this study provides a pragmatic way to help GPs to do this.

## Trial registration number

ClinicalTrials.gov NCT02378779, date of registration: 03/02/2015.

## Data

All data generated or analysed during this study are included in this published article and its supplementary information files.

## Availability of data and materials

All data generated or analysed during this study are included in this published article and its supplementary information files.
